# Therapeutic Potential of 1-(2-Chlorophenyl)-6,7-dimethoxy-3-methyl-3,4-dihydroisoquinoline

**DOI:** 10.3390/molecules29163804

**Published:** 2024-08-11

**Authors:** Valeri Slavchev, Vera Gledacheva, Mina Pencheva, Miglena Milusheva, Stoyanka Nikolova, Iliyana Stefanova

**Affiliations:** 1 Department of Medical Physics and Biophysics, Faculty of Pharmacy, Medical University of Plovdiv, 4002 Plovdiv, Bulgaria; vera.gledacheva@mu-plovdiv.bg (V.G.); mina.pencheva@mu-plovdiv.bg (M.P.); iliyana.stefanova@mu-plovdiv.bg (I.S.); 2 Nonlinear and Fiber Optics, Institute of Electronics, Bulgarian Academy of Science, 72 Tzarigradsko Chaussee, 1784 Sofia, Bulgaria; 3 Department of Bioorganic Chemistry, Faculty of Pharmacy, Medical University of Plovdiv, 4002 Plovdiv, Bulgaria; miglena.milusheva@mu-plovdiv.bg; 4 Department of Organic Chemistry, Faculty of Chemistry, University of Plovdiv, 4002 Plovdiv, Bulgaria; tanya@uni-plovdiv.bg

**Keywords:** 3,4-dihydroisoquinoline, contractile activity, isolated smooth muscle tissue, 5-HT, immunohistochemistry

## Abstract

The synthesized compound 1-(2-chlorophenyl) 6-7-dimethoxy-3-methyl-3,4-dihydroisoquinoline (DIQ) was investigated as a biological agent. Its potential to affect muscle contractility was predicted through in silico PASS analysis. Based on the in silico analysis, its capabilities were experimentally investigated. The study aimed to investigate the effects of DIQ on the ex vivo spontaneous contractile activity (CA) of smooth muscle (SM) tissue. DIQ was observed to reduce the strength of Ca^2+^-dependent contractions in SM preparations (SMP), possibly by increasing cytosolic Ca^2+^ levels through the activation of a voltage-gated L-type Ca^2+^ channel. DIQ potently affected calcium currents by modulating the function of muscarinic acetylcholine receptors (mAChRs) and 5-hydroxytryptamine (5-HT) receptors at a concentration of 50 μM. Immunohistochemical tests showed a 47% reduction in 5-HT_2A_ and 5-HT_2B_ receptor activity in SM cells and neurons in the myenteric plexus (MP), further confirming the effects of DIQ. Furthermore, a significant inhibition of neuronal activity was observed when the compound was co-administered with 5-HT to SM tissues. The conducted experiments confirm the ability of the isoquinoline analog to act as a physiologically active molecule to control muscle contractility and related physiological processes.

## 1. Introduction

The chemistry of natural and synthetic isoquinolines is a rapidly expanding area of study. The high prevalence of isoquinoline chemicals in nature and their significant function as biologically active molecules and compounds with useful medical applications all contribute to the ongoing attention [1,2].

Among the known dihydroisoquinolines, substances with cardiovascular activity, such as sympathomimetics, anticonvulsants, anticoagulants, analgesics, anti-inflammatory, and antiviral agents [3,4,5], and medicinal antispasmodics exist [6]. The presence of the isoquinoline ring is preferred as a structural unit for drug design and plays a pivotal role in the development of effective medicinal preparations for preclinical and clinical applications [7,8].

**Figure 1:**
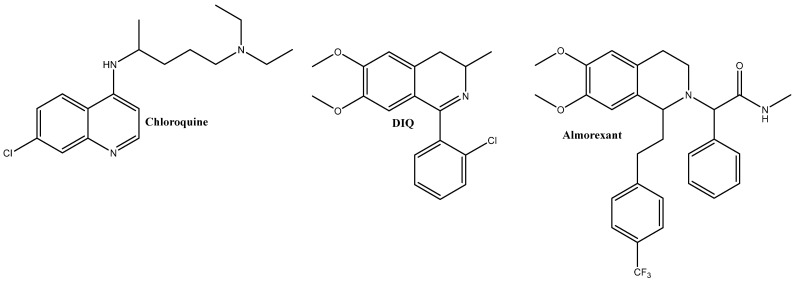
Structures of chloroquine, almorexant, and DIQ.

Previously, the molecule DIQ was synthesized with a very good yield (80%) and subsequently described [9]. The chlorine atom present in well-known drugs such as chloramphenicol, chloroquine, chlorambucil, etc., provides their respective antibacterial, anticancer, and antimalarial properties [10] (Figure 1).

Given the properties that the combined molecule would have from both a chlorine atom and a phenyl ring as functional groups attached to a modified 2-phenylethylamine, DIQ was chosen as a target compound.

The in silico simulations predicted muscle CA for the target molecule DIQ [9]. The results of this analysis can be confirmed experimentally by ex vivo tests on isolated SMPs from the rat stomach in tissue baths and subsequent in vitro histological analyses [10,11,12]. The advantage of this experimental model is that the described processes can modulate cytosolic Ca^2+^ concentration and, therefore, the SMPs’ reactivity to very low doses of the studied substances (up to nM) [13]. SM tissue contractility is regulated by hormones and nerve stimulation is controlled by the autonomic nervous system and local factors [14]. The activation of the SM contractile mechanism is a consequence of changes in the ion channel permeability on the surface of the sarcolemma or changes in the various receptors expressed on its surface.

The current study aims to establish the therapeutic potential of a specific molecule, DIQ, concerning its CA, which is of interest for our studies. 

## 2. Results and Discussion

### 2.1. Ex Vivo Effects of DIQ on Spontaneous CA

The predicted biological activity for DIQ was investigated experimentally by an ex vivo study on isolated circular SMPs in a tissue bath model. This allowed for an analysis of muscle function excluding the influence of other factors. The newly synthesized DIQ was dissolved in DMSO for all biological tests. The solvent induces a slight relaxation effect, the value of which was taken as a baseline for all subsequent effects.

**Figure 2:**
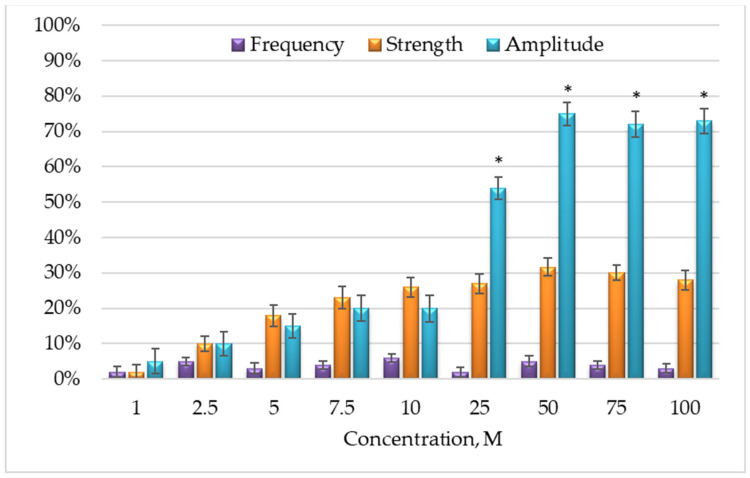
Concentration–effect relationship showing the influence of DIQ on the strength, frequency, and amplitude of spontaneous CA on SMP of the rat stomach. The contraction evoked by 1 μM ACh in the same tissues was found to be 100% (*n* = 16), *—statistically significant differences (*p* < 0.05).

We found that DIQ induced a concentration of SMPs in the range of 1 μM ÷ 100 μM. The influence of DIQ on the strength, frequency, and amplitude of spontaneous CA on the SMP of the rat stomach is shown in Figure 2. The parameters of the spontaneous CA (strength, frequency, and amplitude) were significantly changed for up to 15 min. The maximum tonic contraction appeared after approximately 1.55 ± 0.38 min in all tested concentrations. 

All effects examined were reversible, and the amplitude of these contractions did not change after several applications of DIQ. Each individual application in the tissue bath was separated by changing the Krebs solution with a fresh solution. Its effect generally became significant at a concentration of 25 to 100 μM, when the amplitude was above 50%. The maximal effect of DIQ was observed at a concentration of 50 μM, which was approximately 31.6% of the force of contractions evoked by 1 μM ACh on the same tissues.

The presented results correspond to our previous experiments [15,16,17,18,19], which prove that 1-substituted tetrahydroisoquinolines possess contractile activity and can influence the spontaneous CA of SMP isolated from guinea pigs or rats. The observed CA was not spasmolytic, as usual for this class of compounds. The CA is due to the presence of conjugated double bonds [15,20,21,22].

#### Influence of Specific and Nonspecific Cholinergic Receptor Agonists and Antagonists on DIQ Contractile Effect

Following the treatment of specific receptors in SM cells with biologically active substances, the characteristics and strength of spontaneous contractions were monitored. 

In general, suppressed mACh receptors (mAChR) have an inhibitory effect on Ach release from the presynaptic terminal due to a balance between inhibiting M2/M4 and stimulating their release through M1 receptors. These receptors alternatively influence potassium influx through the presynaptic membrane and intracellular calcium concentration [23]. To establish the role of DIQ in SM contraction processes, we investigated how mAChR agonists and mAChR antagonists affect them. In gastric SM strips experiments, a significant decrease in the size of the contractions was observed in the presence of the mAChR agonist arecoline [24] and the non-selective mAChR antagonists atropine and ipratropium (Table 1). We assumed that the effects of DIQ on the contractile processes are probably caused by a specific triggering action they exert on mAChRs in gastric SM tissue. 

The changes in the strength of DIQ-induced contraction that were observed in the activity of nonspecific muscarinic agonists and antagonists are logical. In both cases, DIQ’s ability to activate all mAChRs was reduced because the isoquinoline molecule interacts with either already activated or already blocked receptors.

**Figure 3:**
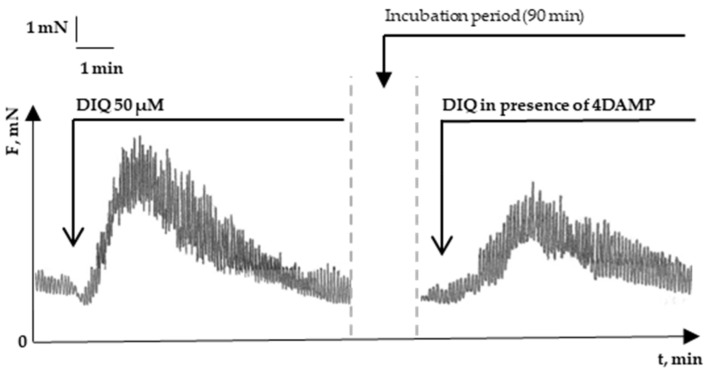
Representative tracings showing the influence of 4-DAMP on the contractile effect of DIQ.

In various gastrointestinal SMs, ACh and its derivatives produce contractions by activating mAChRs. From the family of muscarinic receptors, M1 mAChR, M2 mAChR, and M3 mAChR are expressed predominantly in SM cells [25]. Their activation initiates the processes of Ca^2+^ release from the depots and the activation of L-type Ca^2+^ channels where the cytosolic Ca^2+^ concentration increases, and the contraction process is started. Thus, we have tracked the action of the effectiveness of the selective antagonists for M1 mAChR (dicyclomine and pirenzepine), M2 mAChR (gallamine and alcuronium), and M3 mAChR (4-DAMP and tiotropium) to the DIQ-provoked contractile reaction [26,27,28,29,30]. We found that only with the preliminary M3 mAChR blocking their specific antagonists could a statistically significant decrease in SM response be observed when simultaneously administering DIQ (Table 1, Figure 3). This experimental result indicates that DIQ activity is likely to be related to its influence on intracellular pathways. 

The specific responses observed can be attributed to the parallel pathways induced by DIQ, affecting the contractility of SMPs in the rat stomach. The influence of nicotinic ACh receptors (nAChR) on the DIQ contraction was not considered, since the preliminary blocking of nAChR with hexamethonium and decamethonium (10 μM for the two groups of experiments) did not have a significant effect on the strength of the studied contraction (Table 1). 

Ca^2+^ ions are necessary for contractions and they enter SM cells mainly through voltage-gated Ca^2+^ channels. Thus, to clarify the pathway of DIQ activity more specifically, we decided to apply the nonselective L-type Ca^2+^ channel blocker nifedipine, as well as a selective agent, verapamil, in the tissue baths. The nonselective calcium-blocking agent inhibits SM contractions in various organs, including gastric muscle in vitro [31]. We observed that nifedipine at a concentration of 0.5 μM significantly reduced the strength of the contractile reaction caused by 50 μM DIQ from 2.99 ± 0.17 mN to 0.36 ± 0.06 mN (Table 1). The effect of verapamil at a 0.3 μM concentration is similar—a significant reduction in the contractile response strength from 3.01 ± 0.09 mN to 0.73 ± 0.09 mN. These experimental results indicate that the DIQ contractile effect on SMPs is probably associated with an increase in the cytosolic Ca^2+^ level by activating voltage-gated L-type Ca^2+^ channels [32]. These results undoubtedly confirm our initial hypothesis regarding the pathway used by the DIQ’s CA. 

CA is influenced by different substances that are secreted by neurons in the enteric or autonomic nervous system. Some substances, such as 5-HT, ACh, epinephrine, dopamine, etc., predominantly affect gastrointestinal SMs [33]. ACh is a major neurotransmitter in enteric neurons, which activates the multiple receptors expressed in SM and thus influences SM contraction through the secondary intermediaries IP**_3_** and DAG. On the other hand, activated cytosolic IP**_3_** and DAG initiate some intracellular pathways with the participation of phospholipase C, calmodulin, and myosin light chain protein kinase, thus mediating SM contraction. The neurotransmitter 5-HT influences SM contractility by increasing [Ca^2+^]_i_ via the 5-HT2 receptor subtype, inducing an influx of extracellular Ca^2+^ through L-type voltage-dependent Ca^2+^ channels [34]. Recent studies showed that enteric 5-HT is a polyfunctional signaling molecule that facilitates communication between the enteric nervous system and effector systems (muscles, secretory endothelium, endocrine cells, and the vascular network of the gastrointestinal tract) [35]. The damage and imbalance in serotonergic signaling that influences gastrointestinal motility, secretion, and visceral sensitivity are influenced by defects and deficits in 5-HT production and specific 5-HT receptors [36]. It is known that medicines which target 5-HT signaling molecules are effective in alleviating the symptoms of functional gastrointestinal disturbances. We found that when ACh or 5-HT was exogenously applied in a concentration of 1 μM on SM, a contractile effect with values of 4.91 ± 0.25 mN and 3.88 ± 0.16 mN, respectively, was registered. ACh at the abovementioned concentration applied after DIQ 50 μM does not change the strength of its response, but the strength of the DIQ-induced effect significantly decreases in the presence of 1 μM ACh to 1.15 ± 0.09 mN (Table 2). 

**Figure 4:**
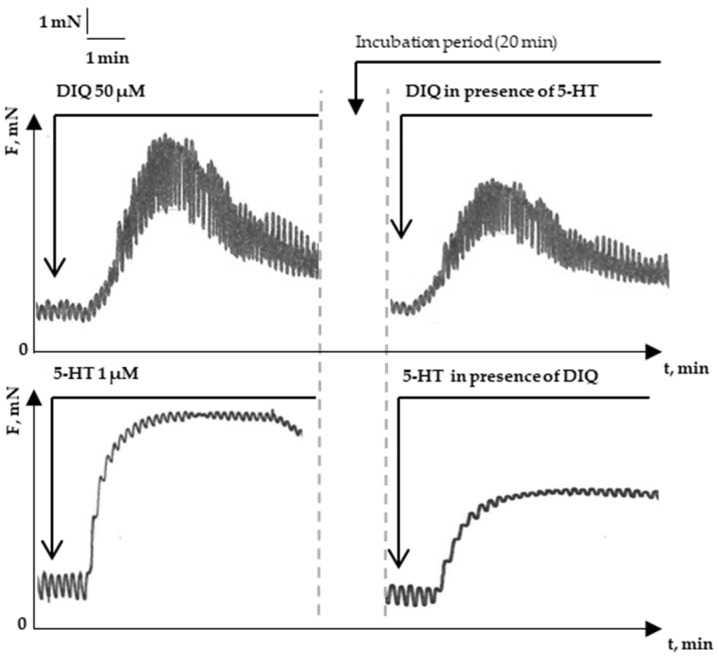
Representative features of SM strip contraction induced by 1 μM 5-HT and 50 μM DIQ under single or combined administration.

In contrast to ACh, after premedication with the other major neuromediator, 5-HT, we found a reduction in the tonic component by 61.5% in the DIQ-induced contractile reaction. The contractile reaction of the neurotransmitter 5-HT in the presence of 50 μM DIQ was also reduced by 60.1% (Figure 4). This mutual influence of the action of 5-HT and DIQ on isolated SMPs experimentally confirms the biological effect predicted in silico which, in this case, is reflected by the change in the physiological functions of the model SM system.

The selected experimental SM model is as simple as possible due to the combined use of isolated tissues and organs from one animal for the SM test and the histological test, respectively.

### 2.2. Histological Determination of the Effect of DIQ on SM

Immunohistochemistry (IHC) localization and registration of the biological effect of DIQ is an optimal instrument for physiological and pharmacological studies aimed at clarifying the mechanisms of action on receptor systems or receptor interactions.

#### 2.2.1. Morphological Analysis

**Figure 5:**
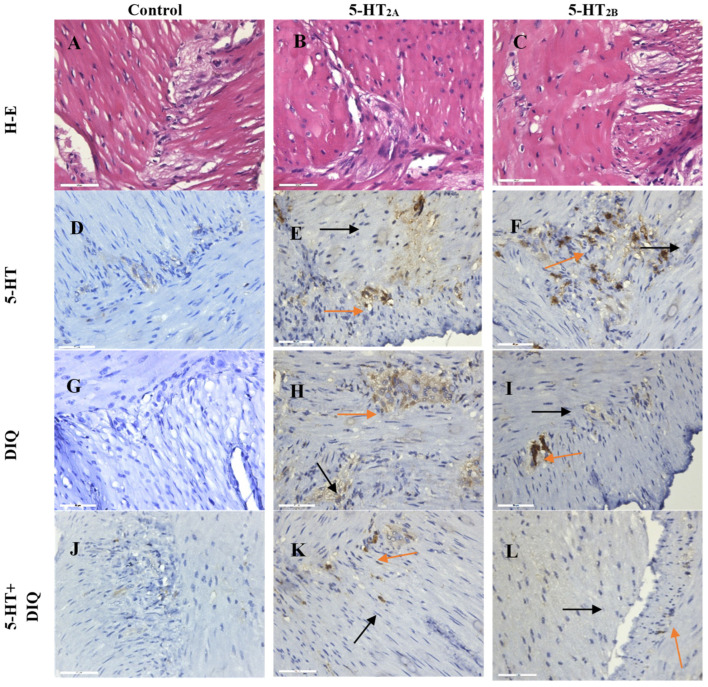
Expression of 5-HT_2A_ and 5-HT_2B_ receptors in gastric preparations (×400 magnification). (**A**) GM preparation incubated with 5-HT, H-E staining; (**B**) incubated with DIQ, H-E staining; (**C**) incubated with 5-HT+DIQ, H-E staining; (**D**) incubated with 5-HT, control; (**E**) incubated with 5-HT, increased intensity, and density in 5-HT_2A_ expression in MP (orange arrows) and SM (black arrows); (**F**) incubated with 5-HT, in presence of 5-HT_2B_ expression in MP (orange arrows) and mild expression in SM (black arrows); (**G**) incubated with DIQ, control; (**H**) incubated with DIQ, a reduced 5-HT_2A_ expression is registered; (**I**) incubated with DIQ, individual cells for 5-HT_2B_ are noticed in the MP(orange arrows) and SM (black arrows); (**J**) incubated with 5-HT+DIQ, control; (**K**) incubated with 5-HT+DIQ, mild 5-HT_2A_ expression is registered in MP (orange arrows) and SM (black arrows); (**L**) incubated with 5-HT+DIQ, reduced intensity in 5-HT_2B_ expression in MP (orange arrows) and SM (black arrows). The incubation period is 20 min.

The histological analysis of SMPs we performed, in which the substance was incubated for 20 min with 5-HT (1 μM), DIQ (50 μM), and a combination of the two substances DIQ+5-HT in their respective concentrations, followed by staining with hematoxylin–eosin (Figure 5A–C), did not register any changes in the two SM layers and the MP between them.

#### 2.2.2. 5-HT_2A_ and 5-HT_2B_ Immune Reaction Determination

IHC analysis indicates an increased expression of 5-HT_2A_ receptors (Figure 5E) and 5-HT_2B_ receptors (Figure 5F) in SM cells and in the MP (Figure 5D) after 5-HT treatment compared to controls. The incubation of tissue preparations in DIQ showed a significant decrease in the expression of 5-HT_2A_ (Figure 5H) and 5-HT_2B_ (Figure 5I) by 43% and 27% respectively, compared to those incubated with 5-HT only (Figure 5E,F). Moreover, we established a reduction in the expression of 5-HT_2A_ (Figure 5K) and 5-HT_2B_ (Figure 5L) by 16% and 12%, respectively, after 5-HT+DIQ combined incubation compared to 5-HT incubation only (Figure 5E,F). A significant reduction in neural activity was registered in the SMPs treated with DIQ alone or in combination with 5-HT. 

**Figure 6:**
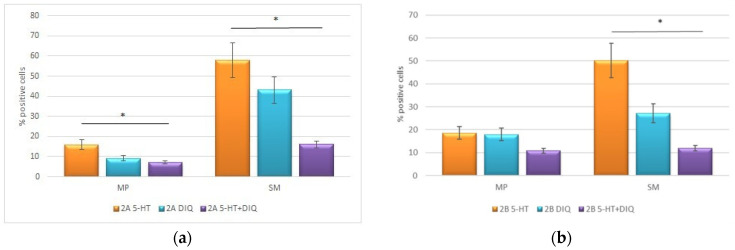
Activity of 5-HT receptors 5-HT_2A_ and 5-HT_2B_ following individual and combined application of 5-HT and DIQ, on rat SM from the stomach and MP. *t*-test was used, where * *p* < 0.05.

The statistical data processing (Figure 6a,b) confirmed the immunohistological observations. The IHC results showed a statistically significant difference between the measurements after the treatment of preparations with 5-HT and DIQ separately, compared to the simultaneous treatment of 5-HT and DIQ. The mean distribution analysis of the results for 5-HT_2A_ and 5-HT_2B_ in MP and SM cells showed a significantly lowered percentage of 5-HT_2A_- (Figure 6a) and 5-HT_2B_- (Figure 6b) positive cells per slice when treated with the combination of 5-HT and DIQ compared to those treated with 5-HT and DIQ separately (*p* < 0.05).

Based on the experimental results observed, we can conclude that the specific biological response of SM cells and neurons in MP in the presence of DIQ is expressed as a significant reduction in the expression of the 5-HT_2A_ and 5-HT_2B_ receptors according to both histological tests conducted. Physiological functions such as contraction and secretion are usually regulated by multiple receptor-mediated mechanisms. Some sequential steps (e.g., receptor binding, multiple intracellular secondary mediators) may intervene between the initial molecular drug-receptor interaction and the final tissue or organ response. Thus, several different pathways can often be used to obtain the same expected response. Our data suggest that the biological effects of DIQ on isolated smooth muscle may be partially caused by a decrease in the density of both membrane serotonin receptors and the associated control of Ca^2+^ influx across the cell surface.

## 3. Materials and Methods

### 3.1. Solutions and Chemicals

The composition of the Krebs solution was as follows (in mM): sodium chloride 120, potassium chloride 5.9, calcium chloride 2.5, magnesium chloride MgCl_2_ 1.2, Sodium Bicarbonate 15.4, monosodium phosphate 1.2, glucose 11.5. The perfusion fluid was aerated with a mixture of O_2_ and CO_2_ (in a ratio of 95%: 5%), and pH was maintained at 7.40. 

The commercially available medicines used were acetylcholine (ACh), 5-HT, nifedipine, verapamil, dicyclomine, alcuronium, arecoline, atropine, ipratropium, pirenzepine, gallamine, tiotropium, 4-diphenylacetoxy-N-methylpiperidine methiodide (4-DAMP methiodide), hexamethonium, and decamethonium. All chemicals and reagents were used without further purification.

Monoclonal anti-SR-2A (A-4) and anti-SR-2B (C-6) antibodies (sc-166775, sc-376878, Santa Cruz, Dallas, Texas, USA, 1:150) were used. IHC staining was performed by using the Autostainer Link 48 (DakoLink IHC Solution, Glostrup, Denmark) with ascending grades of ethanol (70%, 80%, 96%, 100%). 

Concentrated stock solutions of these substances were prepared in a solvent recommended by the manufacturer and aliquots of these stock solutions were added to the tissue bath to obtain the desired concentration. The volume of aliquots was less than 1% of the Krebs solution volume of the tissue bath and was measured continuously with the pH meter HI-5521 (Hanna Instruments Inc., Woonsocket, RI, USA). Deionized water with a conductivity of 18.2 mΩ/cm^2^ was used in all experiments and for the preparation of all necessary solutions. 

### 3.2. Smooth Muscle Preparations

Male Wistar rats with a body weight of 255–285 g (age 10–12 weeks) were used. The animals were observed under standard conditions: room temperature 22 ± 2 °C, free access to water and food and a 12 h dark/light cycle. Two or three circular SM strips were dissected from one rat stomach in situ, separating the muscle tissue and keeping the mucosa intact. The sizes of strips 10–12 mm in length and 1.1–1.3 mm in width, were used to isometrically record the CA. The preparations indicated by *n* were carried out under conditions of continuous irrigation of tissues with a pre-aerated preparation solution containing sodium chloride/potassium chloride/calcium chloride in a ratio of 27.2/1.1/1 with a temperature of 4 °C. 

All experimental procedures were approved by the current European regulations (86/609/EEC) regarding the protection of animals used for experimental purposes, and performed in strict accordance with the current Institutional Animal Care guidelines in Bulgaria and comply with the EU Directive 2010/63/EU.

### 3.3. Registration of the Mechanical Activity of Rat Circular Gastric SM

**Figure 7:**
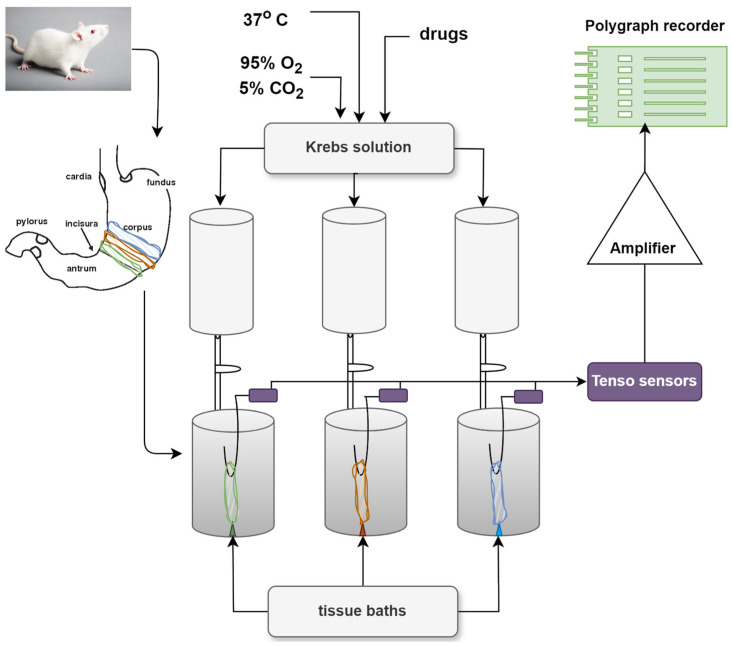
Principle scheme of the experimental setup for recording the mechanical activity of rat circular gastric SM strips in tissue baths.

The CA of SMPs was registered isometrically. The initial mechanical tension of the SMPs was achieved by stretching the tension system with a force of 10 mN. After a 60 min adaptation period, during which the Krebs solution was changed 4 times, and the stabilized spontaneous activity was accepted as the initial tonus. The spontaneous alterations in the mechanical activity and tonus were recorded at this this level utilizing a system of tensodetector (Swema, Stockholm, Sweden) coupled with an amplifier (Microtechna, Prague, Czech Republic) and recorded with polygraph (Linseis, Selb, Germany) (Figure 7). The viability of SMPs was tested by stimulation with a 1 μM ACh neurotransmitter. 

### 3.4. Histological Examination

Stomach tissues were washed with physiological saline solution. They were then placed for 20 min in the isolated tissue bath at 5% CO_2_, 95% O_2_, and 37 °C following the conditions of CA recording experiments. After the incubation time had finished, each SM strip was fixed with 10% neutral formalin.

#### 3.4.1. Hematoxylin-Eosin Staining

After conventional embedding in paraffin wax, 4 serial sections were cut and stained with hematoxylin-eosin for 5 min. Then, slides were dehydrated in a graded ethanol series, cleared with fresh xylene, and mounted onto a slide.

#### 3.4.2. Immunohistochemistry

IHC staining was performed on formalin-fixed and paraffin-embedded 5 μm sections followed by citrate pH 6.0 antigen retrieval, endogenous biotin, and peroxidase blocking. The SM strips were stained for IHC with mouse monoclonal antibodies anti-SR-2A (A-4) and anti-SR-2B (C-6). The images were visualized and captured with a digital camera mounted on a Nikon Eclipse 80i microscope using NIS-Elements Advanced Research Software version 4.13 (Nikon Instruments; Tokyo, Japan). Photomicrographs were observed at a magnification of ×400.

#### 3.4.3. Quantitative Analysis of IHC Reactions

The values were obtained after analysis by groups (*n* = 6) per SM strip. Cells expressing SR-2A and SR-2B in the SM cells and MP of the stomach were analyzed. A grid (19 × 25 fields) was used, through which the number of cells expressing SR-2A/SR-2B marker was determined, according to the formula *x* = (*n*/475) × 100, where *n* is the number of squares in which there are positive cells for SR-2A/SR-2B, and 475 is the total number of squares.

### 3.5. Statistics

The experimental results are presented as the mean ± standard error of the mean (SEM). The number of tissue preparations used in each experiment is denoted by *n*. Statistical differences were tested using the Student’s *t*-test. The probability of less than 5% (*p* < 0.05) was considered significant. Data analysis was performed using the statistical software SPSS, version 16.0 (SPSS Inc. Chicago, IL, USA).

## 4. Conclusions

The present work showed the ex vivo contractile activity of DIQ. The main reason for such behavior is the presence of conjugated double bonds and their ability to activate mAChRs and 5-HT receptors. The experimental results indicated that the specific biological activity of DIQ is significantly reduced in the presence of initially administered receptor agonists, antagonists, and neurotransmitters. 

In the SMPs incubated in DIQ, a significant decrease in the expression of 5-HT_2A_ and 5-HT_2B_ was observed during the IHC analysis. A tendency for the expression of both receptors to decrease when the combination of DIQ and 5-HT is applied is reported. The specific biological response of SM cells and neurons in MP in the presence of DIQ is expressed as a significant reduction in the expression of both serotonin receptors—5-HT_2A_ and 5-HT_2B_. Based on the experimental results, we can conclude that DIQ is a polyfunctional signaling molecule that affects both SM and MP.

## Figures and Tables

**Table 1:** Changes in the tonus of SMPs under the influence of DIQ. The comparison is between DIQ tonic effects in Krebs solution (benchmark) and the presence of selective mAChR and nAChR agonists or antagonists; *n*—number of SMPs; *—statistically significant differences (*p* < 0.05).

SMP Tonus Caused by DIQ (50 μM) (Autocontrol), mN	Background Agent, μM	Time of Incubation of Background Agent, min	Changes in the Tonus of SMPs on the Background Agent, mN	*n*	*p*
3.40 ± 0.31	arecoline	15	1.99 ± 0.19 *	11	0.04
	(0.1 μM)				
3.23 ± 0.16	atropine	30	1.23 ± 0.13 *	11	0.03
	(10 μM)				
3.42 ± 0.20	ipratropium	30	1.67 ± 0.08 *	11	0.01
	(1 μM)				
3.50 ± 0.12	dicyclomine	30	3.48 ± 0.17	10	0.05
	(10 μM)				
3.43 ± 0.10	pirenzepine	90	3.29 ± 0.09	10	0.06
	(10 μM)				
3.48 ± 0.21	gallamine	90	3.08 ± 0.20	10	0.06
	(10 μM)				
3.37 ± 0.22	alcuronium	90	3.12 ± 0.26	10	0.07
	(10 μM)				
3.09 ± 0.08	4-DAMP	90	1.72 ± 0.11 *	10	0.04
	(0.3 μM)				
3.39 ± 0.18	tiotropium	90	1.91 ± 0.14 *	10	0.03
	(5 μM)				
3.22 ± 0.24	hexamethonium	30	3.02 ± 0.12	9	0.05
	(0.1 μM)				
3.34 ± 0.28	decamethonium	30	3.31 ± 0.20	9	0.06
	(0.1 μM)				
2.99 ± 0.17	nifedipine	35	0.36 ± 0.06 *	12	0.01
	(0.5 μM)				
3.01 ± 0.09	verapamil	15	0.73 ± 0.09 *	12	0.01
	(0.3 μM)				

**Table 2:** Mutual influence on contractile activity caused by DIQ on rat circular gastric SMPs and the application of exogenous neurotransmitters ACh and 5-HT: *—*p* < 0.05. *n*—the number of SMPs used in the experiments.

Tonus of SMPs Caused by the Impact Agent (Autocontrol), mN	Background Agent, μM	Time of Incubation of Background Agent, Min	Changes in the Tonus of SMPs on the Background Agent, mN	*n*	*p*
DIQ (50 μM)	ACh (1 μM)	15	1.15 ± 0.09 *	16	0.01
2.36 ± 0.12					
DIQ (50 μM)	5-HT (1 μM)	15	0.99 ± 0.17 *	16	0.03
2.57 ± 0.10					
ACh (1 μM)	DIQ (50 μM)	20	4.85 ± 0.24	18	0.64
4.87 ± 0.25					
5-HT (1 μM)	DIQ (50 μM)	20	2.46 ± 0.23 *	18	0.04
3.88 ± 0.16					

## Data Availability

The original contributions presented in the study are included in the article, further inquiries can be directed to the corresponding authors.
